# Early Response of *Sulfolobus acidocaldarius* to Nutrient Limitation

**DOI:** 10.3389/fmicb.2018.03201

**Published:** 2019-01-10

**Authors:** Lisa F. Bischof, M. Florencia Haurat, Lena Hoffmann, Andreas Albersmeier, Jacqueline Wolf, Astrid Neu, Trong Khoa Pham, Stefan P. Albaum, Tobias Jakobi, Stefan Schouten, Meina Neumann-Schaal, Phillip C. Wright, Jörn Kalinowski, Bettina Siebers, Sonja-Verena Albers

**Affiliations:** ^1^Molecular Biology of Archaea, Institute of Biology II, University of Freiburg, Freiburg, Germany; ^2^Spemann Graduate School of Biology and Medicine (SGBM), University of Freiburg, Freiburg, Germany; ^3^Center for Biotechnology (CeBiTec), Bielefeld University, Bielefeld, Germany; ^4^Department of Bioinformatics and Biochemistry, Braunschweig University of Technology, Braunschweig, Germany; ^5^Molecular Enzyme Technology and Biochemistry (MEB), Biofilm Centre, Centre for Water and Environmental Research (CWE), University of Duisburg-Essen, Essen, Germany; ^6^Department of Chemical and Biological Engineering, The University of Sheffield, Sheffield, United Kingdom; ^7^Department of Marine Microbiology and Biogeochemistry, NIOZ Royal Netherlands Institute of Sea Research, Den Burg, Netherlands; ^8^Department of Earth Sciences, Faculty of Geosciences, Utrecht University, Utrecht, Netherlands

**Keywords:** Archaea, cell motility, signal transduction, transcription factors, nutrient depletion, stress response

## Abstract

In natural environments microorganisms encounter extreme changes in temperature, pH, osmolarities and nutrient availability. The stress response of many bacterial species has been described in detail, however, knowledge in Archaea is limited. Here, we describe the cellular response triggered by nutrient limitation in the thermoacidophilic crenarchaeon *Sulfolobus acidocaldarius*. We measured changes in gene transcription and protein abundance upon nutrient depletion up to 4 h after initiation of nutrient depletion. Transcript levels of 1118 of 2223 protein coding genes and abundance of approximately 500 proteins with functions in almost all cellular processes were affected by nutrient depletion. Our study reveals a significant rerouting of the metabolism with respect to degradation of internal as well as extracellular-bound organic carbon and degradation of proteins. Moreover, changes in membrane lipid composition were observed in order to access alternative sources of energy and to maintain pH homeostasis. At transcript level, the cellular response to nutrient depletion in *S. acidocaldarius* seems to be controlled by the general transcription factors TFB2 and TFEβ. In addition, ribosome biogenesis is reduced, while an increased protein degradation is accompanied with a loss of protein quality control. This study provides first insights into the early cellular response of *Sulfolobus* to organic carbon and organic nitrogen depletion.

## Introduction

Microorganisms are constantly challenged by changes of temperatures, pH, osmolarities or nutrient limitation in their direct environment. To be able to survive, mechanisms to adapt to or counteract these threats have been developed by microbes. Knowledge on the stress response in Archaea is limited and it is not yet understood how changes in the environment are sensed and processed within the cell. Responses to specific stress sources such as pH, oxygen, osmolarity or temperature have been reported (Maaty et al., 2009; Jia et al., 2015; Buetti-Dinh et al., 2016; Wheaton et al., 2016). For example, a temperature shift from 80 to 90°C leads to transcriptomic changes of 30% of protein coding genes in the thermophilic archaeon *Sulfolobus solfataricus* and a regulatory role of a small heat-shock induced transcriptional regulator was proposed in the heat shock response of *Pyrococcus furiosus* (Vierke et al., 2003; Tachdjian and Kelly, 2006). Phosphate starvation negatively affects growth and leads to adaptations in the cellular proteome of *S. acidocaldarius* and it was proposed that the protein content of the cell is exclusive to certain stress sources (Osorio and Jerez, 1996). Further, altered phosphorylation patterns in proteins were observed under phosphate starvation, proposing a regulatory role in the cellular adaptation to external conditions (Osorio and Jerez, 1996).

While knowledge on nutrient starvation in Archaea is limited, the regulatory events that trigger the stringent response in Bacteria are rather well understood. The stringent response represents an intracellular signaling pathway that ensures cellular survival under limiting conditions and is used by the vast majority of Gram-negative Bacteria (Irving and Corrigan, 2018). In *Escherichia coli*, the alarmone ppGpp (Guanosin-3′,5′-bispyrophosphate) is a global gene expression regulator which binds to the RNAP and targets transcription away from growth-related genes toward stress responsive genes whose promoters are controlled by the general stress response master regulator σ^s^ (Chatterji et al., 1998; Toulokhonov et al., 2001). σ^s^ controls expression of approximately 500 genes, including stasis-, oxidative- and osmotic stress survival genes (Magnusson et al., 2005; Weber et al., 2005; Hengge, 2009). However, σ factors are absent from archaeal genomes and RNAP is recruited to promoter regions by multiple general transcription factors, which are pre-assembled at the archaeal promoter (Burton and Burton, 2014; Blombach et al., 2016). Further, pp(p)Gpp is absent from Eury- and Crenarchaeota, raising the question of how nutrient starvation is sensed and the information processed to induce a cellular response (Cimmino et al., 1993; Cellini et al., 2004).

In the acidophilic crenarchaeon *S. acidocaldarius*, nutrient limitation induces directed cell movement toward more favorable conditions (Lassak et al., 2012; Shahapure et al., 2014). Motility in *S. acidocaldarius* is regulated by nutrient depletion dependent transcription factors, Lrs-like proteins and reversible protein phosphorylation (Reimann et al., 2012; Lassak et al., 2013; Orell et al., 2013; Hoffmann et al., 2016; Haurat et al., 2017; Li et al., 2017). However, so far the general cellular response to organic carbon and organic nitrogen depletion has not been addressed. To gain first insights into *S. acidocaldarius* early starvation response, we performed a comprehensive time course study over 4 h to understand how nutrient depletion affects the global transcriptome and proteome over time.

## Materials and Methods

### *Sulfolobus acidocaldarius* Cultivation and Sampling for Transcriptome and Proteome Analysis

The uracil auxotrophic strain *S. acidocaldarius* MW001 (wild type in this study) was grown aerobically at 75°C in Brock’s basal medium supplemented with 0.1% (w/v) NZ-amine, 0.2% (w/v) dextrin, and 10 μg/ml Uracil at pH 3.5 (Brock et al., 1972; Wagner et al., 2012). The minimal medium according to Brock et al. (1972, modified) contains (amount per liter): 1.3 g (NH_4_)_2_SO_4_, 0.28 g KH_2_PO_4_, 0.25 g MgCl_2_ × 7H_2_O, 0.07 g CaCl_2_ × 2H_2_O, 0.02 g FeCl_2_ × 4H_2_O, 1.8 mg MnCl_2_ × 4H_2_O, 4.5 mg Na_2_B_4_O_7_ × 10H_2_O, 0.22 mg ZnSO_4_ × 7H_2_O, 0.06 mg CuCl_2_ × 2H_2_O, 0.03 mg Na_2_MoO_4_ × 2H_2_O, 0.03 mg VOSO_4_ × 2H_2_O, and 0.01 mg CoCl_2_ × 6H_2_O. Cells were starved as previously described for organic carbon and nitrogen sources (Lassak et al., 2012, 2013; Hoffmann et al., 2016). Briefly, *S. acidocaldarius* MW001 was grown until OD_600_ 0.4–0.5 and subsequently harvested (Rotina 380 benchtop centrifuge, 4400 rpm. 10 min, 70°C) and the supernatant completely removed. The pelleted culture was resuspended in the original volume of 75°C pre-warmed basal Uracil containing Brock medium lacking nutrients (starvation culture). From this cell culture, a T0 reference sample was taken (amounts described below) and kept on ice. Samples were taken from the starvation culture after 0.5, 1, 1.5, 2, and 4 h for transcriptomic (10 ml) and proteomic (50 ml) analysis. Cells were grown in biological triplicates and duplicates of each time point were subjected to transcriptomic and proteomic analysis.

### Growth and Intracellular Acidification Measurements

To compare cell growth and pH in nutrient depletion to nutrient rich conditions, a second culture was treated identical to the starvation culture, but was resuspended in nutrient rich medium. The optical density of both cultures was determined after 0.5, 1, 1.5, 2, and 4 h. The experiment was performed in biological triplicates and each sample was taken in duplicate. Simultaneously, one ml of each time point of the nutrient-depleted and the nutrient rich grown culture was pelleted by centrifugation. The supernatant was thoroughly removed and 50 μl of the supernatant were kept. The cell pellet was resuspended to a theoretical optical density of 5 in H_2_0 supplemented with 50 mM KCl. 5x bromphenol-blue containing SDS-loading dye was added to a final concentration of 1x to the resuspended pellet fraction as well as to the supernatant sample (medium control).

### Nutrient Depletion Survival Assay

To assess the survival rate of cells at each time point during nutrient depletion (0.5, 1, 1.5, 2 and 4 h after nutrient depletion relative to timepoint 0 h), 100 μl of 10^-5^-fold diluted cells per time point were plated on Uracil-containing first-selection plates (Wagner et al., 2012). Plates were incubated at 75°C for 4 days and colonies were counted. The total number of colonies obtained for the time point 0 h reference sample was set to 100% and the percentage of colonies for all other time points was calculated relative to time point 0 h. Data from three independent biological replicates and three technical replicates were analyzed using a paired, two-tailed student’s *t*-test.

### RNA Isolation

RNA was isolated from all samples and replicates with Trizol (Thermo Fisher, Waltham, MA, United States) as described previously (Hottes et al., 2004). The obtained RNA samples were treated with RNase-free DNase (Qiagen) according to the manufacturer’s protocol and purified using ethanol precipitation. Ribosomal RNA was depleted using a RiboZero magnetic kit for bacteria (Illumina, San Diego, CA, United States) with a modified protocol in which only 90 μl magnetic beads were used and for the rRNA removal reaction 1 μg RNA was mixed with 4 μl removal solution in a total volume of 20 μl.

### RNA Sequencing and Data Analysis

Sequencing libraries for all samples and replicates were prepared with the TruSeq^®^ Stranded mRNA HT kit (Illumina) starting with the RNA fragmentation step after elution of precipitated RNA in 19 μl of the Fragment-Prime-Mix. Sequencing libraries were quantified with a High-Sensitivity Chip on a Bioanalyzer (Agilent, Böblingen, Germany) and a measurement with a Quant-iT PicoGreen^®^ dsDNA Assay Kit (Invitrogen, Carlsbad, CA, United States) on a Microplate Reader Tecan Infinite 200 (Tecan, Männedorf, Switzerland). Sequencing was performed on a HiSeq1500 instrument (Illumina) in rapid mode with a read length of 2 × 25nt or on a MiSeq instrument (Illumina) with a read length of 2 × 75nt. Sequencing reads were mapped with Bowtie2 (Langmead and Salzberg, 2012) against the reference genome. Since *S. acidocaldarius* MW001 is a deletion mutant of *S. acidocaldarius* DSM639, the latter was chosen as a reference (*S. acidocaldarius* DSM639, genome size: 2,225,959 nt, RefSeq ID: NC_007181.1). The data was visualized in the software ReadXplorer and mapped reads were counted for each gene (Hilker et al., 2014). For determination of regulated genes, a statistical analysis was performed using DESeq2 (Love et al., 2014). For significance, all genes with an adjusted *p*-value below 0.01 and an at least twofold change in transcription were considered as differentially transcribed.

### Protein Extraction and iTRAQ Labeling

Frozen cells of *S. acidocaldarius* MW001 collected at 0 and 0.5 h, 1.5 and 4 h after starvation were washed with ice-cold water before being re-suspended in 1 ml of protein extraction buffer (05% sodium dodecyl sulfate in 0.5 M triethylammonium bicarbonate, pH 8.5). Cell lysis was performed on ice using an ultra-sonicator (Sonifier 450, Branson) 8 times at 70% duty cycle (45 s of sonication and 45 s pause). Samples were collected by centrifugation at 21,000 *g* at 4°C for 30 min (Heraeus Multifuge, Thermo), supernatants (including soluble and insoluble proteins) were collected and protein concentrations were determined using a Bradford assay (Sigma, United Kingdom). A total of 100 μg proteins from each sample (Two biological replicates for each sampling point) was used for an 8-plex iTRAQ analysis with 8 iTRAQ tags used (113, 114, 115, 116, 117, 118, 119, and 121) (Sciex, United States), and the analysis was performed based on the manufacturer’s instructions. Briefly, the proteins were reduced by 2 μl of 50 mM tris- (2-carboxyethyl) phosphine at 60°C for 1 h and alkylated by 1 μl of 200 mM methyl methanethiosulfonate for 10 min at room temperature before being digested by trypsin MS grade (Promega, United Kingdom) with the ratio of trypsin:proteins 1:20. Digested proteins from different time points were then labeled with iTRAQ reagents as follows 0 h: 113 and 114 (control), 0.5 h: 115 and 116, 1.5 h: 117 and 118, and 4 h: 119 and 121, and incubated at room temperature for 2 h. All labeled peptides were then combined before being dried in a vacuum concentrator (Eppendorf Concentrator 5301, Germany).

### Fractionation of Labeled Peptides

Hydrophilic interaction chromatography (HILIC) was used for cleaning and fractionation of dried iTRAQ labeled peptides, in which peptides were re-suspended in 100 μl of HILIC buffer A [10 mM ammonium formate in 80% acetonitrile (ACN), pH 3.0] prior to loading onto a 4.6 × 200 mm Poly HYDROXYETHYL-A column (5 μm, 200Å, Hichrom Limited, United Kingdom) coupled with an uHPLC 3000 system (Dionex, Germany). An UV detector was used to monitor peptides’ abundance at a wavelength of 280 nm. Peptides were fractionated at a flow rate of 0.5 ml/min using a gradient with HILIC buffer B (10 mM ammonium formate in 5% ACN pH 5.0) composed of 10 min of 2% buffer B before ramping up to 20% of buffer B for 5 min, and to 60% of buffer B for 50 min, then ramped up to 100% of buffer B for 10 min and kept for 10 min, and finally 0% of buffer B for 5 min. Eluted peptides were collected every 2 min, dried in a vacuum concentrator, cleaned using C_18_ spinning tips (Nest Group, United States) and submitted to a mass spectrometer (MS).

### Nano LC-MS/MS Analysis

Cleaned peptides (from different fractions) were selected and re-dissolved in 20 μl of buffer A [0.1% formic acid (FA) in 3% CAN] and combined into six different fractions. 3 μl of sample was withdrawn and submitted onto a Q Exactive^TM^ Hybrid Quadrupole-Orbitrap Mass Spectrometer (Thermo, Germany) coupled with a nano uHPLC 3000 system (Dinonex, United Kingdom) operated at a flow rate of 0.3 μl/min. Peptides were separated using a C_18_ column with a 105 min gradient of buffer B (0.1% FA in 97% ACN) as follows: 3% for 5 min, then ramped up to 10% for 5 min, 50% for 75 min, 90% for 1 min, then kept at 90% for 4 min before ramped back to 3% buffer B for 1 min then maintained at 3% for 14 min. The MS was operated in positive mode with resolutions of full MS and ddMS^2^ set at 60,000 and 15,000, respectively. AGC targets were set at 3.10^6^ and 5.10^4^ for full MS and ddMS^2^, respectively. Maximum IT times were set at 100 and 20 ms for full MS and ddMS^2^, respectively. A full mass scan ranging from 375 to 1500 m/z was applied for MS while the mass scan of 100–1500 m/z was applied for ddMS^2^; default charge state of ion was set to 2.

### Identification and Quantitation of Peptides/Proteins

All raw data files from MS analysis were submitted to MaxQuant version 1.5.3.8 for protein identification against *S. acidocaldarius* MW001 database (consisting of 2.361 entries). Modifications of iTRAQ reagents (on N-terminal and Lysine residue) and MMTS were set as fixed modifications while methionine oxidation was set as variable modification; trypsin digestion used with max missed cleavages of 2; minimum peptide length of 6 and maximum peptide mass of 4600 Da were set; tolerances of 20 and 4.5 ppm were applied for MS and MS/MS, respectively. A False Discovery Rate (FDR) of 0.01 was used for identification of both peptides and proteins; a minimum score of 40 was used for modified peptides.

All detected peptides containing intensities of iTRAQ reagents, from MaxQuant, were then submitted to an in-house proteomic pipeline for quantitation of protein and determination of regulated proteins (Pham et al., 2010; Bewley et al., 2011). Firstly, data imported from MaxQuant was filtered to remove both reversed and potential combination peptides, then proteins identified/quantified by a single peptide were removed before intensities of iTRAQ reporters of peptides being transformed into ln form for further analyses. The quantitation was also done using mean and isobaric corrections. *T*-tests (α = 0.01) were then performed at the peptide level (peptides corresponding to an identified protein) to determine regulated proteins for each phenotype comparison. The mass spectrometry proteomics data have been deposited to the ProteomeXchange Consortium via the PRIDE (Vizcaíno et al., 2016) partner repository with the dataset identifier PXD009111. For full lists of detected peptides and quantified proteins as well as regulated proteins, please see detailed data in the ProteomeXchange identifier PXD00911.

### Determination of Lipid Composition

The biomass was refluxed in 2 mL 1.5 N HCl in methanol (MeOH) and stirred for 3 h. After cooling, the solvent was transferred and the pH was adjusted to 4–5 with 2 N KOH/MeOH. Subsequently, 2 mL dichloromethane (DCM) and 2 mL bidistilled H_2_O was added. The solvents were shaken and centrifuged at 3000 rpm for 2 min. The DCM layer was separated and the MeOH/H_2_O layer was extracted 2x with 2 mL DCM. The DCM layers were combined and dried under a N_2_ flow. The extracts were dissolved in DCM and water was removed with anhydrous Na_2_SO_4_ column and dried under a N_2_ flow. The extracts were dissolved in hexane/isopropanol (99:1, v/v) filtered using a 0.45 μm PTFE filter and analyzed using ultra high performance liquid chromatography (HPLC)/mass spectrometry (MS) as described previously (Hopmans et al., 2016).

### Data Availability Statement

#### Transcriptome

https://www.ncbi.nlm.nih.gov/geo/query/acc.cgi?acc=GSE113716 (Reviewer token: qdwxwsuarfadpcz).

#### Proteome

Data are available via ProteomeXchange with identifier PXD009111 (Username: reviewer04418@ebi.ac.uk Password: WBFsvva6).

## Results

### Experimental Design

*Sulfolobus acidocaldarius* was grown in nutrient rich Brock medium (basal Brock medium supplemented with 0.2% dextrin and 0.1% NZ-amine) to an OD_600_ of 0.4 and subsequently transferred to medium lacking organic carbon and organic nitrogen sources (Brock et al., 1972; Lassak et al., 2012; Wagner et al., 2012). Cells were pelleted by centrifugation and the pelleted culture was resuspended in the original volume of 75°C pre-warmed basal Brock medium lacking organic nutrients (starvation culture). At time point 0 h the reference sample was taken from this starvation culture and kept and processed immediately on ice. The culture was further grown at 75°C and samples were taken after 0.5, 1, 1.5, 2, and 4 h for transcriptomic (10 ml) and proteomic (50 ml) analysis.

**FIGURE 1:**
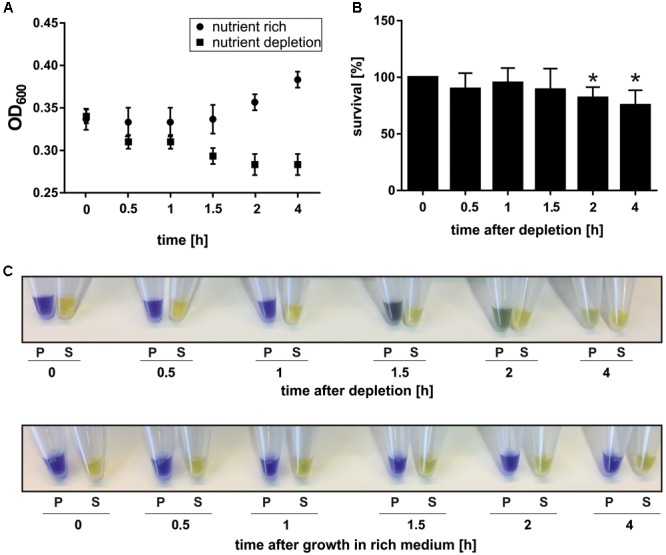
Growth **(A)**, survival **(B)**, and acidification **(C)** of cells exposed to nutrient depletion. **(A)** The OD_600_ was determined over a 4 h time course of nutrient depletion and in a control culture that was re-grown for 4 h in nutrient rich conditions. **(B)** The survival rate of cells exposed to nutrient limitation was determined. A sample from each time point was diluted 10^-5^-fold and 100 μl were plated on nutrient rich plates. After 4 days at 75°C, colonies were counted and the percentage of cell survival relative to time point 0 (100%) was determined. A paired, two-tailed students *t*-test was used to determine the significance. ^∗^ = significant change compared to time point 0, *p*-value < 0.05. Data were obtained from three independent biological replicates performed in triplicate. **(C)** Acidification of whole cells during nutrient depletion. 1 ml of culture was pelleted per time point, the supernatant completely removed and 50 μl kept as medium control. The cell pellet was resuspended in H_2_O supplemented with 50 mM KCl to a theoretical OD_600_ of 5 and SDS-loading dye (contains bromphenol-blue) was added to pellet (P) and supernatant (S) fractions.

The optical density at 600 nm (OD_600_) of the starvation culture and a control culture, which was resuspended after centrifugation in the original volume of 75°C pre-warmed Brock medium supplemented with nutrients (0.2% dextrin and 0.1% NZ-amine), was monitored (Figure 1A). The measured OD_600_ did not increase in nutrient depleted cells, indicating that cells are arrested in growth while the control culture, which was exposed to nutrient rich medium grew normally (Figure 1A) (Wagner et al., 2012). The viability of the nutrient depleted cells during the time course of the experiment was determined using a plating assay. Therefore, cells from each time point were plated on nutrient rich gelrite plates and incubated at 75°C for 4 days (Wagner et al., 2012). Colonies were counted and the survival rate was determined relative to the time point 0 h reference sample. Colonies were obtained for all time points, indicating that *S. acidocaldarius* survives nutrient depletion (Figure 1B). Compared to time point 0 h, a low but significant decrease of survival of approximately 15% was detected after 2 and 4 h of nutrient depletion (Figure 1B). Thus, the majority of cells are viable and retain their ability to grow in nutrient rich conditions after being exposed to depletion conditions for 4 h.

Cell motility of *S. acidocaldarius* is a well-described process that is timely induced on transcript and protein level between 0 and 4 h after exposure to nutrient depletion. This process is orchestrated by a well-described hierarchical signaling network (Lassak et al., 2012, 2013; Reimann et al., 2012; Orell et al., 2013; Hoffmann et al., 2016; Haurat et al., 2017; Li et al., 2017). Therefore, our analysis of physiological, transcriptome and proteome changes in *S. acidocaldarius* was performed over a 4 h time course in which we aimed to understand how the cell responds to nutrient depletion on a more global level than motility regulation. Throughout this study, transcript level changes of genes are written in italics while affected proteins are presented as standard text. All genes with an adjusted *p*-value below 0.01 and an at least twofold change in transcription were considered as differentially transcribed. The cut-off for protein level significance was set to *p*-values from one-paired *t*-tests ≤ 0.01.

### Effects of Depletion on the Cell Membrane and pH Homeostasis

Acidophilic microorganisms have developed mechanisms to maintain a near neutral cytoplasmic pH. These mechanisms include a highly impermeable cell membrane (e.g., tetraether lipid-containing membrane in Archaea), an inside positive membrane potential (ΔΨ), proton transporters from the respiratory chain (Dopson et al., 2002; Baker-Austin and Dopson, 2007), the production of cytoplasmic buffering molecules (basic amino acids and polyamines) and intracellular proton consuming reactions (Ferguson and Ingledew, 2008; Slonczewski et al., 2009). While living in an acidic environment *S. acidocaldarius* maintains an inside pH of 6.5, which is the main contributor to the proton motive force (PMF) and crucial for the energetic status of the cell (Slonczewski et al., 2009).

To test whether *S. acidocaldarius* can keep the cytoplasmic pH constant during the time course of the depletion experiment, cells from the examined time points were pelleted and resuspended in bromphenol-blue containing SDS-loading dye. Interestingly, a color change from blue to yellow of the resuspended cell pellet was observed during the time course suggesting a decrease in pH (Figure 1C). This effect was visible after 1.5 h and increased after 2 and 4 h (Figure 1C). A yellow color, which reflects the pH of the medium of 3.5, was observed upon addition bromphenol-blue containing SDS-loading dye to the supernatant from the all samples (Figure 1C). Therefore, we propose that the cell cytoplasm acidifies during nutrient depletion and investigated changes of membrane properties of cells grown under nutrient rich and depletion conditions.

As depicted in Supplementary Table S4, upon nutrient depletion, the overall amount of cyclopentane rings stayed constant (ring index 3.4) while in the presence of nutrients we observed a distinct shift toward tetraether lipids with a decreased amount of cyclopentane rings (Uda et al., 2004; Zhang et al., 2016). Differences between starved and non-starved cells were found especially in glycerol dialkylglycerol tetraether (GDGT) with -3,-4 and -5 rings. While in the presence of nutrients the percentage of GDGT-3 increased from 11 to 17.5% after 4 h of growth under nutrient rich conditions, it dropped to 9.9% in cells grown under nutrient limitation (Supplementary Table S4). A decrease from 67.6% (0 h) to 56.7% (4 h) was detected for GDGT-4 during nutrient rich growth, while under starvation after 4 h GDGT-4 constitutes still 64% of the membrane. Further, in the presence of nutrients, the overall amount of GDGT-5 dropped from initial 2.4% (4 h) while under nutrient depletion it increased to 3.9% (Supplementary Table S4). The number of cyclopentane rings determines membrane packing and permeability (Gabriel and Lee Gau Chong, 2000; Siliakus et al., 2017). Thus, under nutrient rich growth, membrane permeability increases as a consequence of a decreased number of rings, while under nutrient depletion the overall amount of rings does not change and thus the permeability of the membrane stays constant. In consequence, the membrane is more tightly packed and thus less permeable to protons under nutrient depletion than under normal growth conditions, which indicates that nutrient depletion induces cell envelope changes in *S. acidocaldarius*.

In addition, we detected upregulation of SoxEFGHIM terminal oxidase (described later). This complex translocates two protons. The activity of this complex could also be a mechanism to counteract cytoplasm acidification. To investigate this, we analyzed genes involved in cytoplasmic buffering mechanisms. We observed the upregulation of several permeases and symporters, such as the Na^+^/proline symporter, *saci_0383*. A number of predicted permeases encoding genes of the major facilitator superfamily (MFS) were upregulated at all time points (Supplementary Table S2).

### Global Transcriptome Changes During Nutrient Depletion

**FIGURE 2:**
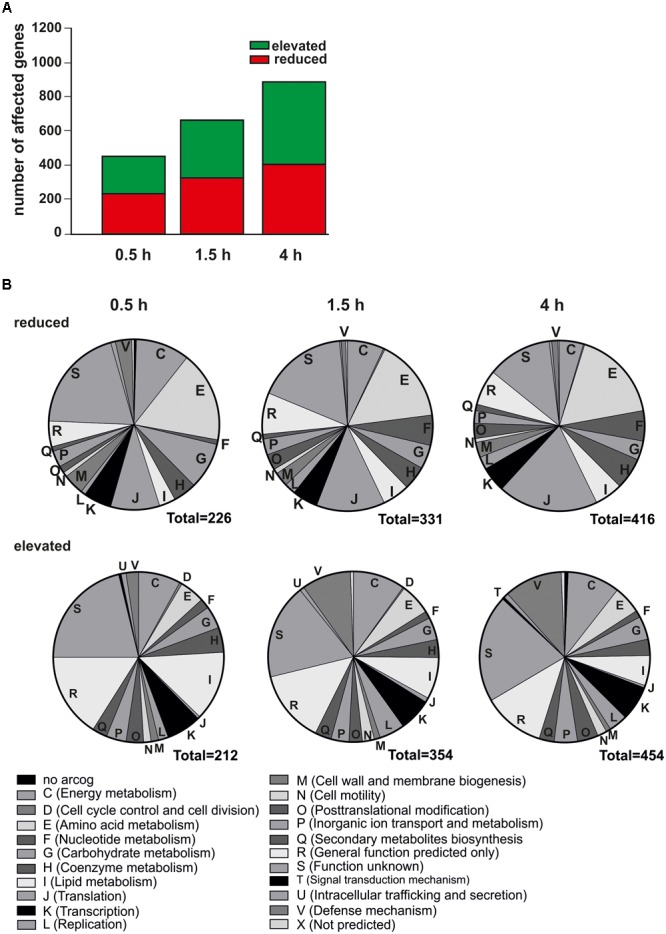
Transcriptomic changes over time in response to nutrient depletion. **(A)** Number of affected transcript levels after 0.5, 1.5, and 4 h of nutrient depletion relative to time point 0 h. Red indicates reduced transcript level of genes, green indicates elevated transcript levels of genes compared to time point 0 h **(B)** Functional classification of affected genes in their respective arCOGs [archaeal cluster of orthologous genes (Wolf et al., 2012)]. Reduced transcript levels (upper) and elevated transcript levels (lower) of genes after 0.5 h (left), 1.5 h (middle), and 4 h (right) of nutrient depletion.

Transcription of approximately 50% of protein-coding genes (1118 of 2223 genes) was affected by nutrient depletion (Figure 2A). After 0.5 h, 438 genes changed their transcription patterns (reduced transcript levels for 212 genes and elevated transcript levels for 226 genes). After 1.5 h 685 genes revealed changed transcript levels (transcript levels of 354 genes elevated and of 331 genes reduced) (Figure 2), peaking in a total number of 870 altered transcript after 4 h (elevated transcript levels for 454 genes and reduced transcript levels for 416 genes) (Figure 2). The transcript levels of 17 genes oscillated and both, elevated as well as reduced transcript levels were found relative to time point 0 h during the course of starvation (Supplementary Table S1). Transcript levels of genes whose products function in a variety of cellular processes were affected by nutrient depletion (Figure 2B). Unfortunately, the majority of genes were of unknown function as assigned to function unknown (S) or general function predicted (R) arCOG [archaeal cluster of orthologous genes (Wolf et al., 2012)], which hampers their functional analysis. Transcripts of multiple genes associated to metabolic arCOGs [i.e., energy metabolism (C), carbohydrate metabolism (G), nucleotide metabolism (F), inorganic ion transport and metabolism (P)] were affected in response to nutrient depletion indicating that the lack of nutrients lead to alterations and rerouting of metabolic pathways (Figure 2B). A detailed pathway analysis is described later. In general, transcript levels of genes assigned to translation (J) were reduced during nutrient depletion, while transcript levels of genes whose products are involved in transcription (K) show a differential regulation. Elevated transcript levels were found for the majority of genes assigned to lipid metabolism (arCOG I), indicating alterations in the cellular membrane during starvation. Further, as reported previously, transcript levels of genes encoding for motility related proteins (arCOG N) were elevated already after 0.5 h of starvation (Lassak et al., 2012, 2013; Hoffmann et al., 2016; Haurat et al., 2017). Apart from that, transcript levels of defense mechanism genes (arCOG V), such as genes encoding for CRISPR related proteins were elevated under nutrient depletion (Figure 2B).

### Global Proteome Changes During Nutrient Depletion

**FIGURE 3:**
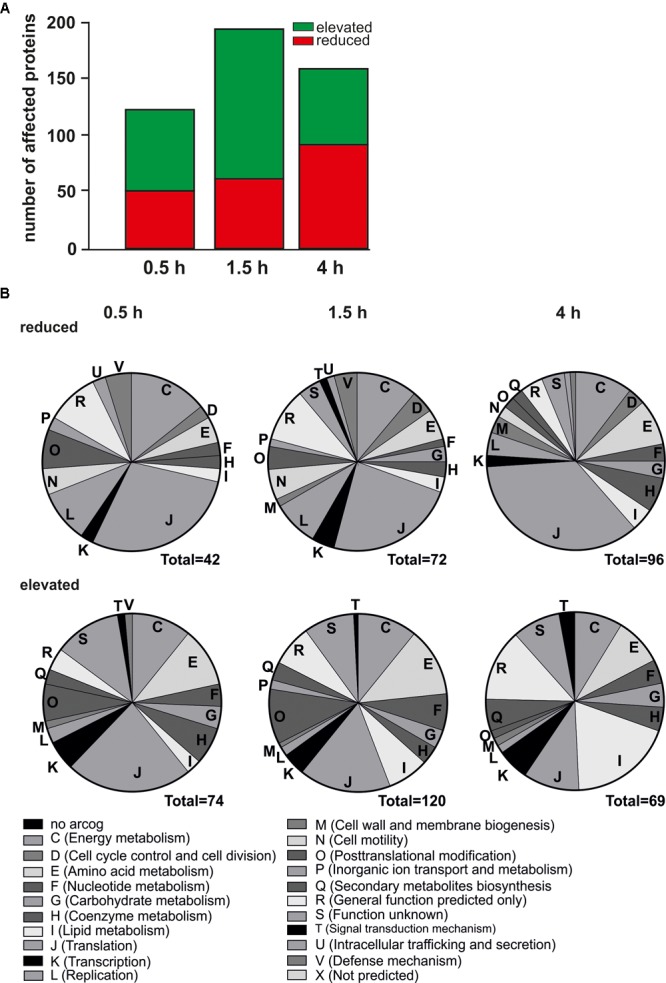
Proteomic changes over time in response to nutrient limitation. **(A)** Number of affected protein levels after 0.5, 1.5, and 4 h of nutrient depletion relative to time point 0 h. Red indicates reduced protein levels, green indicates elevated protein levels. **(B)** Functional classification of affected proteins in their respective arCOGs. Reduced protein levels (upper) and elevated protein levels (lower) after 0.5 h (left), 1.5 h (middle), and 4 h (right) of nutrient depletion.

Next, we investigated changes in the proteome after nutrient depletion using iTRAQ (Figure 3). A total of 19,058 unique peptides corresponding to 1,578 proteins were detected and quantified and the amount of depletion responsive proteins was determined. The total number of proteins affected by starvation increased from 0.5 to 1.5 h and decreased at 4 h (Figure 3A). As shown in Figure 3B, relative protein levels of amino acid metabolism proteins (arCOG E) were severely affected. In agreement with the transcriptome analysis, elevated protein levels were found for lipid metabolism related proteins (arCOG I), proteins required for post-translational modification (arCOG O) and biosynthesis of secondary metabolites (arCOG Q). Relative protein levels of proteins assigned to arCOG J (translation) were affected in both directions, indicating acceleration of translation in the initial phases of nutrient depletion, as most aminoacyl-tRNA synthetases showed the highest upregulation at 0.5 and 1.5 h. Protein levels of ribosomal proteins were reduced after 4 h (Figure 3B, discussed later).

The changes observed at transcript and protein level show that many biological processes are affected by nutrient depletion in *S. acidocaldarius*, which will be described in more detail below.

### Changes of the Central Carbohydrate Metabolism During Nutrient Depletion

**FIGURE 4:**
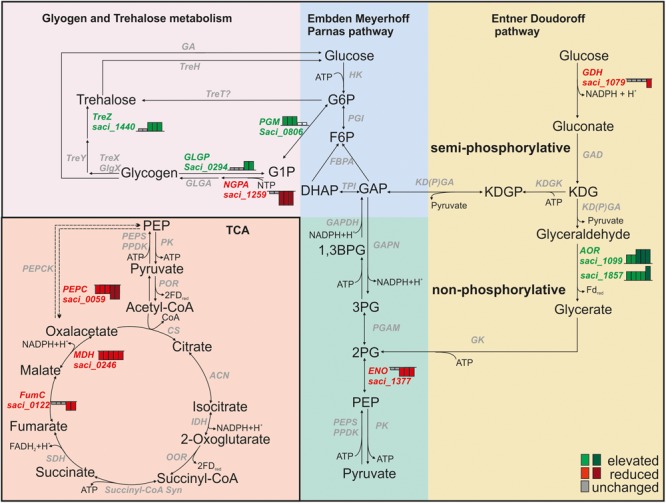
Transcript levels of central carbohydrate metabolism genes in *Sulfolobus acidocaldarius*. Height of Bars next to affected genes depict the respective fold change of transcript levels after 0.5, 1, 1.5, 2, and 4 h. Red indicates reduced transcript levels, green indicates elevated transcript levels, gray indicates basal levels compared to time point 0 h (log2 onefold: lighter colors, log2 twofold and higher: darker colors).

Glycogen is a stable, easily accessible carbon and energy storage compound (Brasen et al., 2014). Transcript levels of genes whose products are involved in formation and degradation of glycogen were inversely affected during depletion (Figure 4). Transcript levels of sugar-1-phosphate nucleotidylyltransferases encoding genes [NTP-glucose-1-phosphate nucleotidylyltransferase (NGPA); *saci_1259, saci_0422*], which are required for the formation of activated sugars (NDP-glucose) from G1P are reduced under starvation, indicating that glycogen synthesis is decreased (Figure 4 and Supplementary Table S2). Transcript levels of enzymes required for degradation of glycogen via G1P to glucose 6-phosphate (G6P), such as the GLGP (*saci_0294*) and the PGM (*saci_0806*) were elevated (Figure 4 and Supplementary Table S2). Increasing the degradation of glycogen allows *S. acidocaldarius* to quickly release glucose intermediates. In addition, glycogen provides a source for trehalose synthesis via the TreY/TreZ pathway. Here, TreY (maltooligosyltrehalose synthase, *saci_1436*) catalyzes the formation of maltooligosyltrehalose. This is followed by the activity of maltooligosyltrehalose trehalohydrolase TreZ (*saci_1440*) and results in trehalose release (Maruta et al., 1996; Schiraldi et al., 2002). Trehalose serves as a compatible solute in *Sulfolobus* spp. and in our analysis elevated transcript levels of *treZ* were detected (Figure 4 and Supplementary Table S2) (Martins et al., 1997). In *S. acidocaldarius* glucose is degraded via the branched Entner Doudoroff (ED) pathway (Kardinahl et al., 1999). In the ED pathway, GDH (*saci_1079*, downregulated after 4 h) catalyzes the initial oxidation of glucose to gluconate. Transcript levels of the AOR [*saci_1099* (Kardinahl et al., 1999)] which catalyzes the oxidation of glyceraldehyde to glycerate are increased during nutrient depletion. Its homolog *saci_1857* (physiological function unknown) follows the same pattern (Figure 4 and Supplementary Table S2). Further, transcript levels of the ENO encoding gene *saci_1377*, which catalyzes the conversion PEP to 2-phosphoglycerate, are reduced from 1.5 to 4 h of nutrient depletion (Figure 4 and Supplementary Table S2).

### Tricarboxylic Acid (TCA) Cycle

*Sulfolobus acidocaldarius* obtains energy by aerobic respiration and complete oxidation of organic compounds via the oxidative TCA cycle forming reduced electron carriers [i.e., NAD(P)H, Fd_red_ and FADH_2_]. Transcript levels of genes encoding phosphoenolpyruvate carboxylase (PEPC; *saci_0059*), the MDH (*saci_0246*) and the FumC (*saci_0122*) were reduced during depletion (Figure 4 and Supplementary Table S2). The PEPC from *S. acidocaldarius* has an anaplerotic role in replenishing the TCA cycle with oxaloacetate. The enzyme is regulated by allosteric inhibition via L-aspartate and L-malate (Sako et al., 1997; Ettema et al., 2004). In contrast to the observations made on transcript level we observed a higher protein level of TCA cycle proteins, such as Saci_0122 (FumC), Saci_0246 (MDH), Saci_0982 (SDH) and Saci_2375 (aconitase ACN) (Supplementary Table S3).

### Respiratory Chain

**FIGURE 5:**
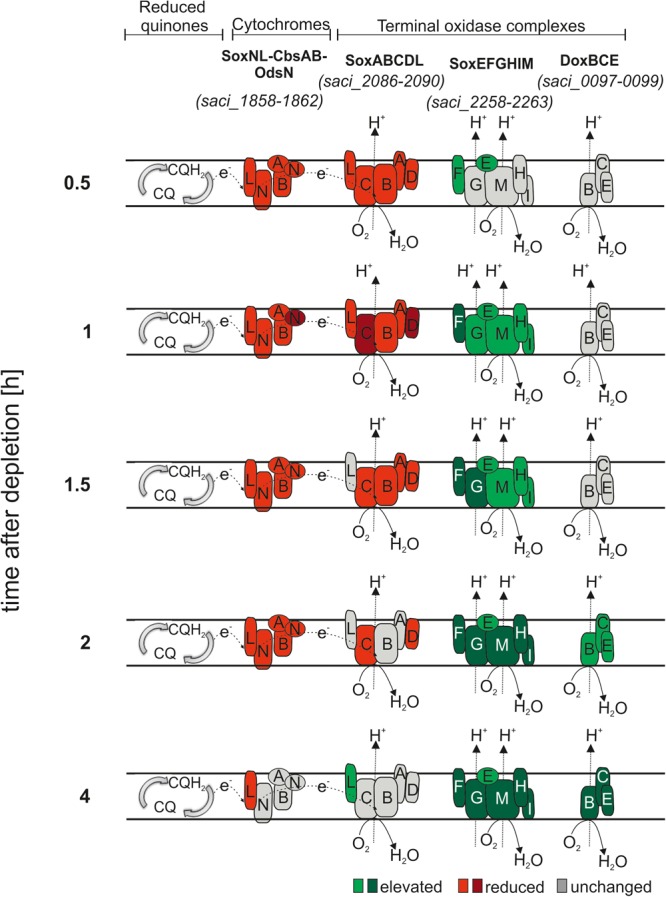
Transcript levels of the proposed aerobic respiratory chain protein coding genes of *S. acidocaldarius* during depletion. Immediately after the cells are shifted to nutrient depletion, transcript levels of the cytochrome oxidase bc1 complex (SoxNL-CbsAB-OdsN) and the terminal oxidase SoxABCDL are reduced (red) and after 2 h several subunits return to their basal level (gray). Inversely, elevated transcript levels are found for the terminal oxidase SoxEFGHIM under nutrient depletion (green). Elevated transcript levels of the DoxBCE complex are found after 2 h ours. CQ, caldariellaquinol. log2 onefold: lighter colors, log2 twofold and higher: darker colors.

Organisms can regulate the energy conservation efficiency according to their needs by simultaneously expressing several terminal oxidases. The *S. acidocaldarius* respiratory chain consists of NADH dehydrogenase, SDH and cytochromes (Lübben et al., 1992). Electrons from NADH and SDH complexes reduce quinones (CQ), which are first delivered to the cytochrome bc1 complex SoxNL-CbsAB-OdsN (Wakao et al., 1987; Moll and Schafer, 1991; Hiller et al., 2003) and subsequently to one of the three terminal oxidases, SoxABCDL (Lübben et al., 1992, 1994; Gleißner et al., 1997; Hiller et al., 2003), SoxEFGHIM (Lubben et al., 1994; Castresana et al., 1995; Komorowski et al., 2002) and DoxBCE (Purschke et al., 1997) (Figure 5). Finally, the electrons are transferred to O_2_, generating a pH gradient coupled to ATP synthesis (Moll and Schäfer, 1988). As depicted in Figure 5, SoxABCDL and DoxBCE pump protons with a different H^+^/e^-^ ratio than SoxEFGHIM (Gleißner et al., 1997; Komorowski et al., 2002). SoxEFGHIM has a lower redox potential than SoxABCD which impacts the electron donor options (Gleißner et al., 1997; Komorowski et al., 2002). It is unknown why several terminal oxidases are present in *S. acidocaldarius*. Each oxidase complex could provide an advantageous adaption system to respond to environmental changes such as pH, temperature, substrate availability or oxygen tension (Lubben et al., 1994; Simon et al., 2009). Therefore, we analyzed the effect of nutrient depletion on their transcript levels. Transcript levels of six NADH dehydrogenase encoding genes (*saci_2338 - saci_2343*) and *SoxNL-CbsAB-OdsN* and *SoxABCDL* were reduced (Figure 5 and Supplementary Table S2). Transcript levels of *SoxEFGHIM DoxBCE* were elevated during depletion (Figure 5 and Supplementary Table S2).

### Glutamate and Polyamine Metabolism

**FIGURE 6:**
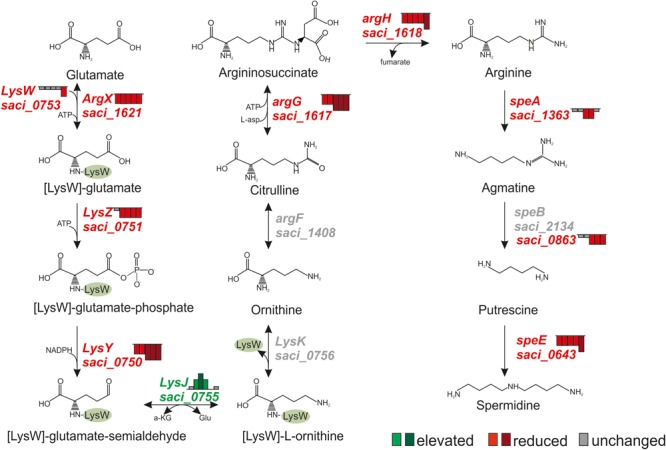
Transcript level changes of genes whose products function in the polyamine synthesis pathway in *S. acidocaldarius*. Height of Bars next to affected genes depict the respective fold change of transcript levels after 0.5, 1, 1.5, 2, and 4 h. Red indicates reduced transcript levels, green indicates elevated transcript levels, gray indicates basal levels compared to time point 0 h (log2 onefold: lighter colors, log2 twofold and higher: darker colors).

Polyamines, such as spermidine and putrescine are well known cytoplasmic buffering components that accumulate in Archaea and Bacteria to counteract cytoplasm acidification (Ferguson and Ingledew, 2008; Slonczewski et al., 2009). Arginine is a well-known precursor for spermidine synthesis in *Sulfolobus* spp. and recently, the lysine and arginine synthesis pathways of *S. acidocaldarius* were characterized (Ouchi et al., 2013). The pathway for spermidine and putrescine from glutamate biogenesis was reconstituted in *S. solfataricus* and the respective homologs were identified in *S. acidocaldarius* using bioinformatics (Esser et al., 2013). As shown in Figure 6, transcript levels of several genes involved in arginine, spermidine and putrescine synthesis were affected during depletion. In agreement with the observed downregulation of the transcriptional activator *lysM* (Saci_0752, see below) the transcript levels of all genes of the arginine biosynthesis pathway were decreased with the only exception of *lysJ* (*saci_0755*) (Figure 6 and Supplementary Table S2). Further, transcript levels of *argG* (*saci_1617*, argininosuccinate synthase), *argH* (argininosuccinate lyase), *speA* (*saci_1363*, arginine decarboxylase), *speB* [*saci_0863* (arginase family enzyme)] and *speE* (*saci_0643*, Spermidine synthase) were reduced (Supplementary Table S2 and Figure 6). However, proteome studies revealed an increased abundance of LysZ (Saci_0751) and ornithine carbamoyltransferase (ArgF; Saci_1408), suggesting that the transcriptional changes are not manifested at protein level.

### Translation Machinery and Protein Quality Control

Transcript levels of translation initiation factors were decreased (*alF2y*/*saci_0832* after 2-4 h, alF1/*saci_0964* after 1.5-4 h, *ABCE1*/*saci_0672* after 2–4 h and *alF5A*/*saci_1311* after 2–4 h) or not affected by starvation (Supplementary Table S2). On the proteome level we observed reduced protein levels for alF2y after 0.5 h and elevated protein levels of Saci_695 (alF5B) after 0.5-1.5 h and alF5A after 1.5 h (Supplementary Tables S2, S3). In addition, many ribosomal proteins of the 30S and 50S ribosomal subunits were mostly downregulated on transcript – and less abundant on protein level (Supplementary Tables S2, S3).

Archaeal ubiquitin-like (Ubl) modifier proteins are covalently attached to various targets (urmylation) and target them for degradation in the proteasome (Anjum et al., 2015). Transcript levels of proteasome subunits (*saci*_*0613* and *saci*_*0909*) as well as the proteasome assembly chaperone PAC2 (*saci_0658*) were decreased during nutrient depletion (Supplementary Table S2). Thus, as a consequence of nutrient depletion protein synthesis as well as protein quality control seem to be reduced or shifted toward synthesis of proteins with essential function in cellular survival.

### Glycosylation

Many archaeal extracellular proteins are *N*-glycosylated and this modification is important for the stability of the cell envelope (S-layer) (Jarrell et al., 2014). As depicted in Supplementary Table S2, transcript level of genes whose products function in the *N*-glycosylation pathway were decreased upon nutrient depletion [*aglH* (*saci_0093), agl2* (*saci_0422)*, aglB (*saci_1274*)] (Gavel and von Heijne, 1990; Meyer et al., 2011, 2017; Meyer and Albers, 2014). Increased transcript levels were found for an asparagine amidase encoding gene (*saci_1148*), whose product is presumably removing asparagine-linked chains from glycoproteins and glycopeptides (Supplementary Table S2). This might constitute a recycling mechanism for glycans. In addition, multiple transcript levels of genes encoding for glycosyltransferases family 2 (*saci_0124, saci_0956, saci_1011*) were decreased at all time points (Supplementary Table S2). These findings suggest that nutrient depletion induces cell envelope changes in *S. acidocaldarius*.

### Transcriptional Regulation of the Archaellum Operon During Nutrient Depletion

**FIGURE 7:**
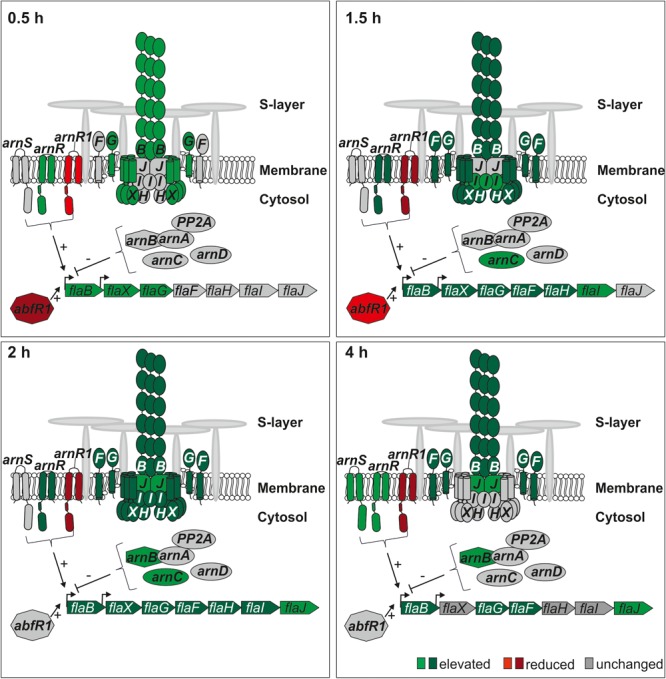
Transcript levels of archaellum proteins and archaellum regulators during nutrient depletion. Red indicates reduced transcript level of genes, green indicates elevated transcript levels of genes compared to time point 0 h. (log2 onefold: lighter colors, log2 twofold and higher: darker colors). Transcripts of archaellum component encoding genes are elevated or not regulated, while elevated and reduced transcript levels of archaellum regulator encoding genes were found.

Motility is the only well-described physiological process that is regulated in response to nutrient limitation in *S. acidocaldarius* (Lassak et al., 2012, 2013). *S. acidocaldarius* uses a type IV pilus like structure, the archaellum, for motility. The archaellum of *S. acidocaldarius* is composed of seven essential proteins (FlaB, FlaX, FlaG, FlaF, FlaH, FlaI, and FlaJ) transcribed from an operon controlled by two promoters (p*flaB* and pf*laX*) (Lassak et al., 2012). The analysis of the regulation of archaellum transcripts in this study was in accordance with earlier results (Lassak et al., 2012, 2013; Hoffmann et al., 2016; Haurat et al., 2017; Li et al., 2017) and thereby validated our current study (Figure 7 and Supplementary Table S2). Unfortunately, archaellum forming proteins were not found in our proteome analysis, which is presumably caused by their membrane and extracellular localization. However, the appearance of *fla* gene transcripts aligned well with earlier Western Blot studies on the expression of FlaB and FlaX (Hoffmann et al., 2016). Transcription of the operon is regulated by the archaellum regulatory network (Arn). The activity of several positive and negative transcription regulators of the Arn depends on reversible phosphorylation by the eukaryotic-like protein kinases ArnC, ArnD, and ArnS (Reimann et al., 2012; Hoffmann et al., 2016; Haurat et al., 2017). In the presence of nutrients, transcription of the archaellum operon is repressed by the ArnA-ArnB complex (Reimann et al., 2012). In this study elevated transcript levels of *arnB* and *arnC* were found after 2–4 h and 1.5 h, respectively (Figure 7 and Supplementary Table S2). Elevated transcript levels of the major transcriptional activator of the archaellum operon, *arnR*, was found, reaching its maximum at 1.5–2 h after nutrient depletion, confirming earlier results obtained by qRT-PCR (Haurat et al., 2017) (Figure 7 and Supplementary Table S2). ArnR senses a so far unidentified signal, binds to the *flaB* promoter and induces operon transcription (Lassak et al., 2013). In contrast, transcript levels of *arnR1*, encoding an ArnR homolog, were strongly reduced at all time points (Supplementary Table S2). Transcript levels of the Lrs14-type transcriptional biofilm and motility regulator *abfR1* were reduced during the initial phases (time points 0.5–1.5 h) of nutrient depletion (Figure 7 and Supplementary Table S2). We observe increased transcript levels of the membrane-bound sensor kinase *arnS* at 4 h after nutrient depletion (Figure 7 and Supplementary Table S2) (Haurat et al., 2017). The hierarchal regulation by the Arn leads to a timed expression of the archaellum. Already 0.5 h after starvation, the transcript levels of the filament forming protein *flaB*, and the scaffold forming protein *flaX* as well as the stator component *flaG* are increased (Figure 7 and Supplementary Table S2). After 1 h, transcript levels of the stator component *flaF* as well as the archaellum clock protein *flaH* are elevated (Figure 7 and Supplementary Table S2). Simultaneously, transcript levels of the archaellum motor ATPase *flaI* are elevated after 1.5 and 2 h of starvation and transcript levels of the membrane protein *flaJ* increase after 2 and 4 h (Figure 7 and Supplementary Table S2). Finally, a functional archaellum is assembled, which starts to rotate and mediates cellular motility (Lassak et al., 2012; Hoffmann et al., 2016; Haurat et al., 2017).

### Transcript Levels of Transcription Factors and Regulators Are Affected by Nutrient Depletion

The archaeal transcription machinery resembles those of eukaryotes while archaeal transcription factors are homologous to those of bacteria and therefore bacterial-type regulators communicate with a eukaryotic-like machinery (Langer et al., 1995; Bell and Jackson, 1998; Ouhammouch and Geiduschek, 2005). Archaea encode general transcription factors (GTFs) which are homologs of the eukaryotic TATA box binding protein (TBP), the transcription factor TFIIB (TFB) and the transcription factor E (TFIIE in Eukaryotes, TFE in Archaea) (Facciotti et al., 2007). However, in contrast to Eukaryotes most Archaea encode multiple GTFs. *S. acidocaldarius* encodes for one TBP (*saci_1336*), three TFBs [TFB1 (*saci_0866*), TFB2 (*saci_1341*) and TFB3 (*saci_0665*)] and one TFE with α- and β- subunit (*saci_0652* and *saci_1342*, respectively) (Bell and Jackson, 2000; Blombach et al., 2015; Feng et al., 2018; Schult et al., 2018). Upon nutrient depletion, transcript levels of *TFB1, TFB2*, and *TFEβ* were elevated (Supplementary Table S2) whereas the transcription of *TBP, TFB3*, and *TFEα* was not affected. Unfortunately, these proteins were not found in our proteome analysis.

In addition to the GTFs, transcript and protein levels of several bacterial-type transcriptional regulators were affected by nutrient depletion. The transcriptional regulators of the Lrp/Asn family represent important regulators of amino acid metabolism and pili synthesis in Bacteria and homologs are also found in Archaea (Peeters and Charlier, 2010). In *S. acidocaldarius*, transcript levels of different Lrp/Asn family type transcriptional regulators were affected by nutrient depletion (Supplementary Table S2) such as *saci_0092, saci_0731* (reduced transcript levels) and *saci_0944, saci_1658* (elevated transcript levels) (Supplementary Table S2). Elevated transcript as well as protein levels were found for Saci_1588 (Supplementary Tables S2, S3). Two Lrp-type amino-acid metabolism regulators were affected by nutrient depletion. The lysine responsive Lrp-type regulator LysM (*saci_0752*) activates transcription of the lysine biosynthesis operon and enhances lysine production under normal growth conditions (Brinkman et al., 2002). Reduced transcript levels of *lysM* were found from 1 h after nutrient limitation. Elevated transcript levels were found for BarR (*saci_2136*), a well-known activator of an aminotransferase (*saci_2137*, elevated transcript levels after 0.5 and 1 h) which is involved in degradation of β-alanine to acetyl-CoA and CO_2_ (Liu et al., 2014, 2016).

The Lrs14 family of transcriptional regulators is distantly related to the Lrp/Asn family and represents an archaea-specific family of small DNA-binding proteins (Pérez-Rueda and Janga, 2010). *S. acidocaldarius* harbors at least six Lrs14-like proteins of which five are affected by nutrient depletion on transcript and protein level (Supplementary Tables S2, S3) (Orell et al., 2013). Of these six regulators, the archaellum regulator AbfR1 is the best studied Lrs14 regulator (see previous section) (Li et al., 2017). Elevated transcript levels were found for *saci_1242, saci_0133*, and *saci_1219* and also protein levels of Saci_1242 and Saci_1219 show higher abundance of the regulators during nutrient depletion (Supplementary Tables S2, S3). Reduced transcript and protein levels were found for *saci_1223* (Supplementary Tables S2, S3).

In addition, several predicted transcription regulators that contain helix-turn-helix-domains were affected by depletion on transcript as well as protein level (Supplementary Tables S2, S3).

In summary, we observed significant changes at transcript and protein levels of GTFs as well as different types of transcriptional regulators upon nutrient depletion, which makes them likely candidates for the regulation of the starvation response in *S. acidocaldarius*.

## Discussion

In this work we analyzed the early response of *S. acidocaldarius* to nutrient depletion. After growth in complex media (dextrin and NZ-amine), organic carbon and organic nitrogen sources were removed and the cellular response was followed by transcript- and proteomics over a 4 h time course.

Although we observed major changes at the transcript and protein levels, the overlap between both data sets was low. In previous studies in *S. solfataricus* where we analyzed the effect of growth on different carbon sources we observed a rather specific response with a good overlap between both data sets (Wolf et al., 2016; Stark et al., 2017). However, also from other studies as for example in *Thermoplasma thermophilus* (Sun et al., 2010), there is evidence that the response to stress conditions is much more complex and different levels of post-transcriptional and post-translational modification are involved.

With the limitation of energy, amino acids and nucleotides, the cell has to optimize gene expression and protein synthesis to survive starvation. In Bacteria, the cellular response is well studied and in *E. coli* the sigma factor RpoS and the stringent response have been identified as important global control systems (Magnusson et al., 2005; Battesti et al., 2011). In *S. acidocaldarius* we observed significant changes in the transcript levels of the general transcription factors TFB2 and TFEβ upon starvation. For TFB2, a cell-cycle dependent expression has been shown with induction upon transition from the G1 to the S-phase and a role in cell cycle regulation was proposed (Lundgren and Bernander, 2007). TFEβ stimulates transcription by stabilizing formation of the preinitiation complex and enhancing DNA melting (Blombach et al., 2015). Previous studies demonstrated that TFEβ is depleted in the stationary phase and upon oxidative stress (H_2_O_2_) leading to downregulation of TFEαβ dependent promoters and reprogrammed transcription in response to different growth phases (Blombach et al., 2015). Therefore, the observed upregulation upon depletion confirms an important regulatory role of TFEβ in transcription regulation similar to sigma factors in Bacteria. In addition, we observed numerous transcriptional regulators that were differentially regulated and probably regulate more specific cellular responses.

During depletion, the cell is forced to re-direct its metabolism to supply biosynthetic precursors as well as energy in order to maintain the central cellular functions. *Sulfolobus* spp. and acidophiles in general have to preserve the cellular neutral pH and counteract acidification. Our data indicate that glycogen as internal carbon storage is degraded and either glucose-6 phosphate or trehalose [via the TreY/TreZ pathway (Maruta et al., 1996; Schiraldi et al., 2002)] are formed. Apart from its function as a compatible solute and in general stress response, as described for many bacteria (Strøm and Kaasen, 1993) (Martins et al., 1997), recent reports about trehalases in *Sulfolobales* suggest also an important function of trehalose as carbon source (Moon et al., 2016; Lee et al., 2018). Trehalose provides D-glucose, which can be catalyzed via the branched ED pathway. In addition, there is evidence that the cell reduces the *N*-glycosylation of S-layer proteins and removes and degrades glycans to provide additional carbon sources, which aids survival of environmental nutrient limitation.

With the depletion of dextrin and NZ-amine in the growth medium and thus depletion of accessible amino acids the cell is forced to synthesize amino acids using the inorganic nitrogen provided in the growth medium. However, this is energetically costly and requires active protein synthesis. Our data indicate that *S. acidocaldarius* responds by protein degradation, reduced protein quality control and reduced translation. The latter is reflected by a significant decrease in ribosomal proteins and translation initiation factors, which reflects active ribosomes.

For *Sulfolobus* spp. one of the greatest challenges during nutrient depletion is the maintenance of the pH gradient (pH ∼3 outside of the cell and pH ∼6.5 inside of the cell), which is directly coupled to ATP synthesis via the PMF (proton motive force). During early nutrient depletion, *S. acidocaldarius* can use the ΔpH to produce ATP “for free,” but risks acidification of the cytoplasm. Therefore, *S. acidocaldarius* cells try to keep the pH homeostasis by switching to terminal oxidase complexes which transport more protons per translocation cycle (Figure 5) and by keeping the number of cyclopentane rings in the lipids high (Supplementary Table S4) as this decreases the proton permeability of the membrane (Gabriel and Lee Gau Chong, 2000; Siliakus et al., 2017). However, after 4 h of depletion these strategies seem to fail as the acidification of the cells was observed and the ΔpH cannot be used for energy conservation anymore. Therefore, with the assembly of the archaellum *S. acidocaldarius* decides to swim to more favorable conditions.

Notably, only few of the changes we observed at the transcriptome level overlap with a previous study where the effect of osmotic and pH response in *S. acidocaldarius* was studied, suggesting that nutrient depletion has a different effect on the cellular response (Buetti-Dinh et al., 2016). Thus, we compared the changes observed during starvation to changes of genes that were recently described for osmotic or pH stress responses in *S. acidocaldarius* (Buetti-Dinh et al., 2016). For our analysis, we considered the changed transcription of genes when *S. acidocaldarius* cells were grown in optimal conditions and shifted to stressful conditions [starvation, pH (acidification) or reduced salt (potassium and/or sodium)] (Buetti-Dinh et al., 2016). Only 27 genes are likewise regulated exclusively under osmotic stress and starvation and seven genes are similar regulated in all three stress conditions (Supplementary Table S5). Therefore, starvation presents a specific challenge for the cell that requires major metabolic as well as cellular changes with tight control of transcription and translation for cell survival.

## Author Contributions

LH, MFH, and JW grew cells and took samples. AA, TP, and JW generated raw data. AA, JW, MN-S, and AN analyzed parts of the data and wrote parts of the manuscript. SA and TJ contributed to transcriptome data analysis. TP, PW, and JK analyzed the data. LB and MFH combined all data and wrote the manuscript. SS performed the lipid analysis. BS and S-VA coordinated the study and wrote the manuscript.

## Conflict of Interest Statement

The authors declare that the research was conducted in the absence of any commercial or financial relationships that could be construed as a potential conflict of interest.

## Abbreviations

1,3BPG: 1,3-bisphosphoglycerate
2PG: 2-phosphoglycerate
ACN: aconitase
AOR: aldehyde oxidoreductase
argF: ornithine carbamoyltransferase
argG: argininosuccinic synthase
argH: argininosuccinic lyase
CS: citrate synthase
DHAP: dihydroxyacetonephosphate
ENO: enolase
FBP: fructose 1,6-bisphosphate
FBPA/ase: fructose-1,6-bisphosphate aldolase/phosphatase
FumR: fumarase
G1P: glucose 1-phosphate
GAA: glucan-1,4-α-glucosidase
GAD: gluconate dehydratase
GAPDH: glyceraldehyde-3-phosphate dehydrogenase
GAPN: non-phosphorylating glyceraldehyde-3-phosphate dehydrogenase
GDH: glucose dehydrogenase
GK: glycerate kinase
GLGA: glycogen synthase
GLGP: glycogen phosphorylase
GlgX: glycogen debranching enzyme
HK: hexokinase
IDH: isocitrate dehydrogenase
KD(P)GA: 2-keto-3-deoxy-(6-phospho)gluconate/2-keto-3-deoxy-(6-phospho)galactonate aldolase
KD(P)Gal: 2-keto-3-deoxy-6-(phospho)galactonate
KDGK: 2-keto-3-deoxygluconate kinase
LysJ: [LysW]-glutamate semialdehyde transaminase
LysK: [LysW]-ornithine hydrolase
LysY: [LysW]-L-glutamate phosphate reductase
LysZ: [LysW]-Glutamate-kinase
MDH: malate dehydrogenase
NGPA: NTP-glucose-1-phosphate nucleotidyltransferase
OOR: α-ketoglutarate dehydrogenase
PEP: phosphoenolpyruvate
PEPC: phosphoenolpyruvate decarbocylase
PEPCK: phosphoenolpyruvate carboxykinase
PEPS: phosphoenolpyruvate synthetase
PGAM: phosphoglycerate mutase
PGI: glucose 6-phosphate isomerase
PGK: phosphoglycerate kinase
PGM: phosphoglucomutase
PHI/HPS: 3-hexulose-6-phosphate isomerase/3-hexulose-6-phosphate synthase
PK: pyruvate kinase
POR: pyruvate ferredoxin oxidoreductase
R5P: ribulose 5-phosphate
SDH: succinate dehydrogenase
speA: arginine decarboxylase
speB: agmatine ureohydrolase/polyamine synthase
speE: aminopropyl transferase
TK: transketolase
TPI: triosephosphate isomerase
TPP: trehalose-6-phosphate phosphatase
TPS: trehalose-6-phosphate synthase
TreH: trehalase
TreX: debranching enzyme
TreY: maltooligosyltrehalose synthase
TreZ: malotoligosyltrehalose hydrolase.ysW, carrier protein, argX [LysW]-Glutamate-ligase
