# Supplementary material for: Early Response of Sulfolobus acidocaldarius to Nutrient Limitation
Source: Front Microbiol. 2019 Jan 10;9:3201. doi: 10.3389/fmicb.2018.03201 (PMC6335949; doi:10.3389/fmicb.2018.03201)
Supplement: Supplementary file 1 [file Data_Sheet_1.PDF]

Supplementary Material

Early response of *Sulfolobus acidocaldarius* to nutrient limitation

Lisa F. Bischof, M. Florencia Haurat , Lena Hoffmann, Andreas Albersmeier, Jacqueline Wolf, Astrid Neu , Trong Khoa Pham, Stefan P. Albaum[3], Tobias Jakobi[3], Stefan Schouten, Meina Neumann-Schaal[4], Phillip C Wright, Jörn Kalinowski, Bettina Siebers & Sonja-Verena Albers*

***correspondence:** sonja.albers@biologie.uni-freiburg.de

Table S1 Genes with oscillating transcript levels during nutrient depletion. Changes after 0.5 h, 1 h, 1.5 h, 2 h and 4 h are depicted. Red indicates reduced transcript levels, green indicates elevated transcript levels, grey indicates basal transcript levels compared to time point 0 h (log2 1-fold: lighter colors, log2 2-fold and higher: darker colors).

**Locus**	**Function**	**0.5 h**	**1 h**	**1.5 h**	**2 h**	**4 h**
Saci_0155	gdhA, OB-fold domain and Zn-ribbon containing protein, possible acyl-CoA-binding protein					
Saci_0815	Acetyl-CoA acetyltransferase					
Saci_1360	Uncharacterized membrane protein					
Saci_1361	acaB1, Glutamate dehydrogenase/leucine dehydrogenase					
Saci_1763	appC, ABC-type dipeptide/oligopeptide/nickel transport system, ATPase component					
Saci_1764	ABC-type oligopeptide transport system, ATPase component					
Saci_1765	Uncharacterized membrane protein					
Saci_1766	Rieske Fe-S protein					
Saci_2090	Uncharacterized membrane protein, SoxL					
Saci_2091	Uncharacterized component of anaerobic dehydrogenase					
Saci_2092	Fe-S-cluster-containing dehydrogenase component					
Saci_2100	Pyruvate:ferredoxin oxidoreductase or related 2-oxoacid:ferredoxin oxidoreductase, beta subunit					
Saci_2101	HerA helicase					
Saci_2197	ABC-type dipeptide/oligopeptide/nickel transport system, permease component					
Saci_2198	TusA-related sulfurtransferase					
Saci_2252	porA, Rhodanese-related sulfurtransferase					
Saci_2253	porB, ferredoxin oxidoreductase, alpha subunit					

Table S2 Transcript levels of genes regulated during nutrient depletion that were analyzed in this study. Changes after 0.5 h, 1 h, 1.5 h, 2 h and 4 h are depicted. Red indicates reduced transcript levels, green indicates elevated transcript levels, grey indicates basal transcript levels compared to time point 0 h (log2 1-fold: lighter colors, log2 2-fold and higher: darker colors).

	Locus	Function	0.5h	1 h	1.5 h	2 h	4 h
**Transcription regulators**	*saci_0866*	Housekeeping general transcription factor TFB1					
	saci_1341	Transcription initiation factor TFB2					
	saci_1342	RNA polymerase III subunit, TFEβ					
	saci_0092	geranylgeranyl pyrophosphate synthetase					
	saci_0731	Lrp/AsnC family C-terminal domain					
	saci_0752	Lrp-like transcriptional regulator LysM					
	saci_0944	Lrp/AsnC family C-terminal domain					
	saci_1658	Lrp family transcriptional regulator,					
	saci_1588	transcriptional regulator					
	saci_2136	transcriptional regulator AsnC family, BarR					
	Saci_2137	aminotransferase,					
	saci_2040	Lrp family transcriptional regulator					
	saci_1342	Lrp/AsnC family					
	saci_1979	Transcriptional regulator, ArsR family					
	saci_0449	SRSR binding protein					
	saci_1242	Lrs14 type regulator					
	saci_0133	Lrs14 type regulator					
	saci_1219	Lrs14 type regulator					
	saci_1223	Lrs14 type regulator					
	saci_2167	archaeal HTH DNA binding domain protein					
	saci_2103	Transcriptional regulator					
	saci_2116	archaeal HTH DNA binding domain protein					
	saci_2296	archaeal HTH DNA binding domain protein					
	saci_0355	Lrp family transcriptional regulator					
	saci_1523	Transcriptional regulator					

**Archaellum & regulators**	*saci_1211*	archaellum repressor ArnB					
	saci_1193	protein kinase, ArnC					
	saci_1178	archaelluin, flaB					
	saci_1177	stator, flaX					
	saci_1176	flaG					
	saci_1175	flaF					
	saci_1174	FlaH					
	saci_1173	Aaa-Atpase, FlaI					
	saci_1172	membrane protein FlaJ					
	saci_0446	Lrs14 type regulator abfR1					
	saci_1181	protein kinase, ArnS					
	saci_1180	archaellum activator, arnR					
	saci_1171	arnR1					
**Central carbon metabolism**	*saci_1259*	Nucleoside-diphosphate-sugar pyrophosphorylase NGPA					
	saci_0294	Glycogen phosphorylase GLGP					
	saci_0806	Phosphomannomutase PGM					
	saci_1440	1,4-alpha-glucan branching enzyme TreZ					
	saci_1079	gdh, dehydrogenase					
	saci_1099	dehydrogenase (AOR)					
	saci_1377	Enolase (ENO)					
	saci_1857	dehydrogenase (AOR)					
**Tricarboxylic acid**	*saci_0059*	Phosphoenolpyruvate carboxylase (PEPC)					
	saci_0246	Malate/lactate dehydrogenase					
	saci_0122	Fumarase (FumC)					
**Polyamine synthesis**	*saci_0751*	LysZ, [LysW]-Glutamat-kinase					
	Saci_0753	LysW carrier protein					
	saci_0750	LysY, [LysW]-Glutamat-phosphat-reductase					
	saci_1621	ArgX, [LysW]-Glutamate-ligase					
	saci_0755	LysJ, [LysW]-Glutamate semialdehyde transaminase					
	saci_1617	Argininosuccinate synthase, argG					
	saci_1618	Argininosuccinate lyase, argH					

	saci_1363	Arginine decarboxylase, speA					
	saci_0863	Arginase family enzyme, speB					
	saci_0643	Spermidine synthase, speE					
**Translation machinery**	*saci_0832*	Translation initiation factor alF2y					
	saci_0964	aIF1A					
	saci_0672	ABCE1					
	saci_1311	AlF5A					
	saci_0081	30S ribosomal protein S4P					
	saci_0571	50S ribosomal protein					
	saci_0575	50s ribosomal protein					
	saci_0576	50S ribosomal protein					
	saci_0577	30S ribosomal protein					
	saci_0578	50s ribosomal protein					
	saci_0579	50s Ribosomal protein					
	saci_0580	50S ribosomal protein					
	saci_0581	50 s ribosomal protein					
	saci_0584	50S ribosomal protein					
	saci_0585	30S ribosomal protein					
	saci_0586	30S ribosomal protein					
	saci_0587	50S ribosomal protein					
	saci_0588	30S ribosomal protein					
	saci_0591	30S ribosomal protein					
	saci_0592	50S ribosomal protein					
	saci_0593	30S ribosomal protein					
	saci_0594	50S ribosomal protein					
	saci_0596	: 5oS ribosomal protein					
	saci_0597	50Sribosomal protein					
	saci_0608	Ribosomal protein					
	saci_0617	50S ribosomal protein					
	saci_0620	30S ribosomal protein					
	saci_0642	Ribosomal protein					
	saci_0697	50 s ribosomal protein					
	saci_0758	30s ribosomal protein					
	saci_0829	30S ribosomal protein					
	saci_0831	30S ribosomal protein					
	saci_0853	Ribosomal protein					
	saci_1469	30S ribosomal protein					
	saci_1520	50S ribosomal protein					
	saci_0613	proteasome alpha subunit					
	saci_0909	proteaseome beta subunit precursor					
	saci_0658	Archaeal enzyme of ATP-grasp superfamily PAC2					
**Cytochrome bc 1 complex**	*saci_1858*	cbsA cytochrome b558/556 subunit A					
	saci_1859	cbsB cytochrome b558/556 subunit B					
	saci_1860	SoxL					

	saci_1861	SoxN cytochrome b					
	saci_1862	OsdN					
**Terminal oxidase complex**	*saci_2086*	soxD					
	saci_2087	SoxC cytochrome b					
	saci_2088	soxB cytochrome c & quinol oxidase polypetide I					
	saci_2089	soxA conserved quinol oxidase polypeptide II					
	saci_2090	soxL					
	saci_2258	SoxI					
	saci_2259	soxH cytochrome c oxidase subunit II					
	saci_2260	soxG cytochrome b					
	saci_2261	soxF conserved archaeal Rieske domain protein					
	saci_2262	soxE sulfocyanin, blue copper protein					
	saci_2263	soxM quinol oxidase polypeptide I/III					
	saci_0097	DoxB cytochrome c oxidase polypeptide I					
	saci_0098	DoxC conserved protein					
	saci_0099	DoxE					
	saci_2338	NADH dehydrogenase					
	saci_2339	NADH dehydrogenase subunit L					
	saci_2340	NADH dehydrogenase subunit k					
	saci_2341	NADH dehydrogenase subunit J					
	saci_2342	NADH dehydrogenase subunit I					
	saci_2343	NADH dehydrogenase subunit H					
**Glycosylation**	*saci_0093*	aglH ,					
	saci_0422	Agl2, Glucose-1-p uridyltransferase					
	saci_1274	aglB,Oligosaccharyltransferase membrane subunit					
	saci_1865	Glycosyl transferase family 2					
	saci_1915	Glycosyl transferase family 2					
	saci_1148	Peptide N-acetyl-beta-D-glucosaminyl asparaginase amidase A					
	saci_0124	Glycosyl transferase family 2					
	saci_0956	Glycosyl transferase family 2					
	saci_1011	Glycosyl transferase family 2					
**major facilitator superfamily**	*saci_0675*	MFS family permease					

Common stress genes	*saci_1059*	MFS family permease					
	saci_1725	MFS family permease					
	saci_1782	MFS family permease					
	saci_1943	MFS family permease					
	saci_1731	MFS family permease					
	saci_2076	MFS family permease					
	saci_1671	MFS family permease					
	saci_0371	MFS family permease					
	saci_1806	MFS family permease					
	saci_1903	MFS family permease					
	saci_2095	MFS family permease					
	saci_1036	ABC-type dipeptide/oligopeptide/nickel transport system, permease component					
	saci_1155	acetate decarboxylase					
	saci_0003	membrane protein					
	saci_0383	Na+/proline symporter					
	saci_1304	Isopropylmalate/homocitrate/citramalate synthase					
	saci_1762	ABC-type dipeptide/oligopeptide/nickel transport system, permease component					
	saci_2317	aapE, ATPase involved in adhesive pili biosynthesis					
	saci_2332	Membrane protease subunit, stomatin/prohibitin homolog					
	saci_0355	transcriptional regulator, Lrp family					
	saci_0094	HAD superfamily hydrolase					
	saci_0394	cobM, Precorrin-4 methylase					
	saci_0925	Hypoxanthine phosphoribosyltransferase					
	saci_1241	HGG motif-containing thioesterase, possibly involved in aromatic compounds catabolism					
	saci_0254	Sec-independent protein secretion pathway component TatC					
	saci_0703	SAM superfamily enzyme					
	saci_0806	Phosphomannomutase					
	saci_2055	decarboxylase					
	saci_2135	RecB family nuclease					
	saci_2137	aminotransferase					
	saci_2225	Acyl-Coa-synthase (AMP-forming)/AMP-acid ligase II					
	saci_2297	Pirin-related protein					

	saci_2306	Pyruvate:ferredoxin oxidoreductase or related 2-oxoacid:ferredoxin oxidoreductase					
	saci_0073	Acetyltransferase (GNAT) family					
	saci_0260	Biotin carboxylase					
	saci_0175	Diphtamide synthase subunit DPH2					
	saci_0435	HerA helicase					
	saci_0620	Ribosomal protein S3AE					
	saci_0750	argC, Acetylglutamate semialdehyde dehydrogenase					
	saci_0832	Translation initiation factor 2, gamma subunit eIF-2 gamma,					
	saci_1523	Transcriptional regulator,					
	saci_1605	GTP-binding protein,					
	saci_1865	Glycosyl transferase family 2					
	saci_1642	tRNA A37 threonylcarbamoyladenosine synthetase subunit TsaC/SUA5/YrdC					
	saci_2111	MFS family permease					
	saci_2200	Uroporphyrinogen-III methylase					
	saci_2267	Rhodanese-related sulfurtransferase					

Table S3 Relative protein levels compared to time point 0 h during nutrient depletion that were analyzed in this study. Changes after 0.5 h, 2 h and 4 h are depicted. Red indicates reduced protein levels, green indicates elevated protein levels, grey indicates basal levels compared to time point 0 h. (log2 1-fold: lighter colors, log2 2-fold and higher: darker colors)

		**Function**	**0.5 h**	**1.5 h**	**4 h**
**Transcriptional regulators**	Saci_1588	transcriptional regulator			
	Saci_0449	SRSR binding protein			
	Saci_1242	Lrs14 type regulator			
	Saci_1219	Lrs14 type regulator			
	Saci_1223	Lrs14 type regulator			
	Saci_2167	HTH DNA binding domain protein			
**Translation machinery**	Saci_0832	Translation initiation factor alF2y			
	Saci_1311	AlF5A			
	Saci_0695	alF5B			
	Saci_0080	30S ribosomal protein			
	Saci_0081	30S ribosomal protein			
	Saci_0082	30S ribosomal protein			
	Saci_0084	50S ribosomal protein			
	Saci_0086	30S ribosomal protein			
	Saci_0571	50S ribosomal protein			
	Saci_0575	50s ribosomal protein			
	Saci_0576	50S ribosomal protein			
	Saci_0577	30S ribosomal protein			
	Saci_0578	50s ribosomal protein			
	Saci_0579	50s Ribosomal protein			
	Saci_0580	50S ribosomal protein			
	Saci_0584	50S ribosomal protein			
	Saci_0585	30S ribosomal protein			
	Saci_0586	30S ribosomal protein			
	Saci_0588	30S ribosomal protein			
	Saci_0591	30S ribosomal protein			
	Saci_0592	50S ribosomal protein			
	Saci_0593	30S ribosomal protein			
	Saci_0594	50S ribosomal protein			
	Saci_0596	50S ribosomal protein			
	Saci_0597	50Sribosomal protein			
	Saci_0603	elongation factor EF2			
	Saci_0617	50S ribosomal protein			
	Saci_0620	30S ribosomal protein			
	Saci_0685	elongation factor IF-2			
	Saci_0688	30S ribosomal protein			

	Saci_0697	50 s ribosomal protein			
	Saci_0758	30s ribosomal protein			
	Saci_0829	30S ribosomal protein			
	Saci_0831	30S ribosomal protein			
	Saci_1458	50S ribosomal protein			
	Saci_1459	50S ribosomal protein			
	Saci_1469	30S ribosomal protein			
	Saci_1495	50S ribosomal protein			
	Saci_1520	50S ribosomal protein			
**Electron donors**	Saci_2343	NADH dehydrogenase subunit H			
	Saci_0979	Succinate dehydrogenase			
	Saci_0982	Succinate dehydrogenase			
**Common stress genes**	Saci_1304	Isopropylmalate/homocitrate/ citramalate synthase			
	Saci_2317	aapE, ATPase involved in adhesive pili biosynthesis			
	Saci_0703	radical SAM superfamily enzyme			
**Central carbon metabolism**	Saci_1816	Glucan 1,4-α-glucosidase (GA)			
	Saci_0619	Sugar phosphate nucleotidyl transferase (NGPA)			
	Saci_0166	aldehyde dehydrogenase (AOR)			
	Saci_0419	BadF-type ATPase, related to human N-acetylglucosamine kinase (FBPA)			
	Saci_0424	KDGK			
	Saci_0837	Phosphoglycerate mutase (PGAM)			
	Saci_1355	pgk ,phosphoglycerate kinase (PGK)			
	Saci_1356	Glyceraldehyde 3-phosphate dehydrogenase (GAPDH)			
	Saci_1965	Alcohol dehydrogenase (GDH)			
**Tricarboxylic acid**	Saci_0122	Fumarate hydratase (FumC)			
	Saci_0246	lactate/malate dehydrogenase (MDH)			
	Saci_0982	sdhA succinate dehydrogenase flavoprotein subunit (SDH)			
	Saci_2375	isocitrate dehydrogenase (ACN)			
**Polyamine**	Saci_0751	[LysW]-Glutamat-kinase, LysZ			
	Saci_1408	Ornithine carbamoyltransferase argF			

Table S4 Percentage of archaeol, GTGT (Glycerol trialkyl glycerol tetraether) and GDGT (Glycerol dialkyl glycerol tetraether with 0-5 cyclopentane rings. GDGT-X marked with ' or '' represent isomeric forms (X= number of cyclopentane rings). Samples were taken in triplicate at T 0 and after 1.5 h and 4 h of starvation (-N) and after 1.5 h and 4 h in the presence of nutrients (+N) (upper part of table) and averaged as well as the ring index were calculated (lower part of table +N = grown in nutrient rich conditions. –N=grown under nutrient depletion.

	Archaeol	GTGT-0	GDGT-0	GDGT-1	GDGT-2'	GDGT-2	GDGT-3'	GDGT-3	GDGT-4'	GDGT-4	GDGT-4''	GDGT-5	Ring index
T 0 h 1.rep	0.2	0.2	4.1	6.8	0.1	6.0	0.4	11.0	1.0	67.3	0.2	2.4	3.4
T 0 h 2.rep	0.2	0.2	4.0	6.7	0.1	5.9	0.4	11.1	0.8	67.7	0.2	2.4	3.4
T 0 h 3.rep	0.2	0.2	4.1	6.7	0.1	5.9	0.4	11.0	0.9	67.7	0.2	2.3	3.4
1.5 h +N 1.rep	0.2	0.3	5.0	6.9	0.2	7.2	0.5	13.8	0.9	62.2	0.2	2.0	3.3
1.5 h +N 2.rep	0.2	0.3	5.5	7.9	0.2	7.7	0.4	14.8	0.8	60.1	0.2	1.8	3.2
1.5 h -N 2.rep	0.1	0.3	4.8	7.6	0.1	6.4	0.5	10.5	1.0	65.1	0.2	3.0	3.4
1.5 h -N 3.rep	0.1	0.3	4.8	7.8	0.1	6.3	0.5	10.6	0.9	65.1	0.2	2.9	3.4
4 h +N 1.rep	0.2	0.2	5.6	8.0	0.2	8.3	0.5	16.9	0.7	57.5	0.2	1.4	3.2
4 h +N 2.rep	0.2	0.2	5.7	8.2	0.2	8.5	0.5	17.7	0.7	56.3	0.1	1.4	3.2
4 h +N 3.rep	0.2	0.2	5.6	8.2	0.2	8.6	0.5	17.7	0.7	56.2	0.2	1.5	3.2
4 h -N 1.rep	0.1	0.3	5.1	7.9	0.2	6.4	0.5	9.7	1.0	64.2	0.3	3.9	3.4
4 h -N 2.rep	0.1	0.3	5.3	8.1	0.1	6.4	0.5	10.0	1.0	63.7	0.3	3.8	3.3
4 h -N 3.rep	0.1	0.3	4.8	7.7	0.1	6.2	0.5	10.1	1.0	64.6	0.3	3.9	3.4

average

T0	0.2	0.2	4.1	6.7	0.1	6.0	0.4	11.0	0.9	67.6	0.2	2.4	3.4
1.5 h +N	0.2	0.3	5.3	7.4	0.2	7.5	0.5	14.3	0.9	61.2	0.2	1.9	3.3
4 h +N	0.2	0.2	5.6	8.1	0.2	8.5	0.5	17.5	0.7	56.7	0.2	1.4	3.2
1.5 h -N	0.1	0.3	4.8	7.7	0.1	6.3	0.5	10.5	1.0	65.1	0.2	3.0	3.4
4 h -N	0.1	0.3	5.1	7.9	0.1	6.3	0.5	9.9	1.0	64.2	0.3	3.9	3.4

Table S5 Genes similarly regulated under starvation and osmotic stress limitation (A. Buetti-Dinh et al, Microbiol (United Kingdom) 162:1422-34, 2016)

**Upregulated genes**	**Downregulated genes**
arnB (*saci_1211*)	*saci_0073* (Acetyltransferase (GNAT) family)
saci_0254 (*tatC*)	*saci_0260* (Biotin carboxylase)
saci_0355 (DNA-binding transcriptional regulator, Lrp family)	*saci_0175* (Diphthamide synthase subunit DPH2)
saci_0703 (Radical SAM superfamily enzyme)	*saci_0435* (HerA helicase)
saci_0806 (Phosphomannomutase)	*saci_0620* (Ribosomal protein S3AE)
saci_1059 (MFS family permease)	*saci_0750* (*argC*)
saci_2055 (3-polyprenyl-4-hydroxybenzoate decarboxylase or related decarboxylase)	*saci_0832* (Translation initiation factor 2, gamma subunit eIF-2 gamma, GTPase)
saci_2135 (RecB family nuclease with coiled-coil N-terminal domain, paREP7 family protein)	*saci_0892* (Uncharacterized Zn-finger containing protein)
saci_2137 (4-aminobutyrate aminotransferase or related aminotransferase)	*saci_1523* (Transcriptional regulator, contains ATP-binding and MarR family HTH domains)
saci_2225 (Acyl-CoA synthetase (AMP-forming)/AMP-acid ligase II)	*saci_1605* (GTP-binding protein, GTP1/Obg family)
saci_2297 (Pirin-related protein)	*saci_1865* (Glycosyl transferase family 2)
saci_2306 (Pyruvate:ferredoxin oxidoreductase or related 2-oxoacid:ferredoxin oxidoreductase, alpha subunit and gamma)	*saci_1642* (tRNA A37 threonylcarbamoyladenosine synthetase subunit TsaC/SUA5/YrdC)
	saci_2111 (MFS family permease)
	saci_2200 (Uroporphyrinogen-III methylase)
	saci_2267 (Rhodanese-related sulfurtransferase)
